# Impact of DMFT, PUFA, DAI, and TDIs on Oral Health-Related Quality of Life (OHRQoL) Among Foundling, Delinquent, and Mainstream School Children: A Prilimenary Study

**DOI:** 10.3389/fpubh.2022.894638

**Published:** 2022-07-08

**Authors:** Abdulaziz Abdullah Alsaif, Thamer Adel Alkhadra, AlBandary Hassan AlJameel

**Affiliations:** ^1^Department of Pediatric Dentistry and Orthodontics, College of Dentistry, King Saud University, Riyadh, Saudi Arabia; ^2^Department of Periodontics and Community Dentistry, College of Dentistry, King Saud University, Riyadh, Saudi Arabia

**Keywords:** dental health surveys, health-related quality of life, oral health, quality of life, children

## Abstract

**Objectives:**

To assess oral health and its implication on oral health-related quality of life (OHRQoL) among groups of foundling and delinquent children compared to mainstream children in Riyadh, Saudi Arabia.

**Materials and Methods:**

This cross-sectional observational study was conducted on children in care houses and mainstream school-going children. The following variables were measured for each group: Demographic data (age, gender); subjective oral health condition; (OHRQoL); clinical oral health condition including the decayed, missing, and filled teeth (DMFT) index; pulpally involved, ulceration, fistula, and abscess (PUFA) index; Dental Aesthetic Index (DAI) for malocclusion, and traumatic dental injuries (TDI).

**Statistical Analysis:**

A one-way ANOVA test, Chi-square test, and Pearson correlation coefficient were used.

**Results:**

The total OHRQoL score was significantly higher for the delinquent compared to the mainstream group. In addition, the DMFT and mean PUFA scores were significantly higher for the delinquent group than the others. The DAI revealed statistical significance in occlusion status within the foundling and delinquent groups, and the prevalence of TDI was significantly higher in the delinquent vs. the mainstream group.

**Conclusion:**

Oral health status appeared to have an association with the OHRQoL among foundling, delinquent, and mainstream children.

## Introduction

Good health has been defined by the World Health Organization (WHO), as “a state of absolute physical, psychological, and social well-being and not just the absence of disease or infirmity” (1). This definition remains the most acceptable definition (2). Oral health, defined as the health of the oral cavity, is known to reflect the general health of an individual (3). Subsequently, a new definition was given by Glick and coworkers for oral health and it contemplates that oral health is multi-faceted and includes the ability to speak, smile, smell, taste, touch, chew, swallow, and convey a range of emotions through facial expressions with confidence and without pain discomfort and disease of craniofacial complex (4). This has a variety of functions in clinical research and surveys (4–7). Social well-being was included in the description of health; the concept of oral health was also become broadened by its inclusion (8). Yewe–Dyer (9) described oral health as a state of the oral cavity and its associated structures wherein disease was contained, and future disease was prohibited, occlusion was adequate for masticating food, and teeth were in a socially acceptable position. Dolan (10) described oral health as a convenient and functional dentition that permits individuals to carry on in their desired social roles. The WHO defines oral health as a state free from any long-standing oral or facial pain, mouth ulcers, cancer in the throat and oral cavity, birth deformities like cleft lip or palate, periodontal diseases, dental caries, teeth loss, or any other condition that affects the oral cavity (11). “Oral diseases” refers to any condition that causes pain, chewing, and appearance problems (12).

For the past decades, dental caries has been assessed globally using DMFT/dmft index (13). However, to quantify the various progressive stages of a carious lesion, a new measuring system was developed the PUFA index (P–Pulpal involvement, U-Ulceration, F-Fistula, and A-abscess) (14). Dental caries, malocclusion is also considered a dental problem since it may lead to undesirable esthetics, speech difficulties, impaired oral function, increased susceptibility to dental trauma, temporomandibular joint disorders, and periodontal diseases (15). Traumatic Dental injuries (TDI) are among the most frequent injuries to the body and have become a matter of concern for public health dentists. They may lead to physical impairment, pain, and emotional distress, which can reduce the social and mental quality of life (16). In 1920, the British economist Arthur Pigou was the first to use the term “Quality of life” (QoL) (17). According to the Center for Health Promotion at the University of Toronto, quality of life is concerned with the extent to which an individual appreciates the significant prospects of life (18). OHRQoL is the personal perception regarding an individual's potential to carry out the essential activities that are influenced by his or her health status (19). Hence, the evaluation of OHRQoL must consider the person's living conditions, cultural background, hopes, and accomplishments (8).

A neglected but essential segment of the community includes foundling and delinquent children and adolescents. “Foundling” refers to a child that has been stranded by his or her parents and under the care of other people (20). In contrast, “delinquent” refers to a child who has broken the law or is involved in the indecent or immoral activity and requires rehabilitation (21). A search for relevant literature revealed that no study had been undertaken to assess the oral health condition of Saudi foundling or delinquent children and its influence on their ORHQoL. According to a data availability study by United Nations, the prevalence was 23.8% in children up to 3 years, while for children aged 3–6, it was 57.3% (22). According to a systematic review (13), the prevalence of dental caries in primary dentition is approximately 80%, and for permanent dentition, it is about 70% in Saudi children (23). The high prevalence of dental caries in Saudi children and adolescents is well documented as is their poor oral hygiene (23). The prevalence of dental caries has been the subject of many studies. Foundling and delinquent children in Riyadh, Saudi Arabia, are an inseparable part of the community, yet a literature search regarding the evaluation of their oral health condition and treatment revealed a scarcity of relevant data. The literature also lacks research into the relationship between ORHQoL and dental decay, malocclusion, and TDI. Thus the present study evaluated oral health and its implications on the OHRQoL among these children compared with mainstream children in Riyadh, Saudi Arabia. Thus, the study aimed to assess the oral health status, including the DMFT and PUFA indices, DAI, and TDIs, and their impact on OHRQoL of foundling and delinquent children compared with mainstream school children.

## Materials Additionally, Methods

The research proposal was submitted to the Institutional Review Board at King Saud University and was given approval number E-19-3797. Legal guardians were requested to sign an informed consent form prior to the recruitment of children for the study, and the oral assent of each child was documented.

### Study Sample and Design

The study was designed as a cross-sectional, observational study involving foundling and delinquent children in care houses and mainstream school children in Riyadh city. Only children were recruited in the study and unwilling children and those who were unable to respond to the study due to mental or physical disability were excluded.

### Variables Measured

Demographic information (age and gender), subjective oral health condition, OHRQoL, clinical oral health condition including the decayed, missing, and filled teeth (DMFT) and pulpally involved, ulceration, fistula, and abscess (PUFA) indices, Dental Aesthetic Index (DAI) index for malocclusion, and TDIs were used for the analysis in the study. A validated Arabic Version of the Child Perception Questionnaire (CPQ) (11–14) was used to determine the OHRQoL (11), in all three groups. This questionnaire was divided into four parts concerning oral symptoms (6), functional impediments (9), emotional well-being (9), and social well-being (12). Each response was scored according to the following rubric: 0, never; 1, once/twice; 2, sometimes; 3, often; and 4, every day. The total of the 36 questions gave a general assessment of the extent of a child's oral health status on his or her quality of life. The highest and lowest possible scores for the total scale were 144 and 0, respectively. The principal investigator filled in the questionnaire through an interview with the child and kappa statistics showed an excellent intra examiner reliability (*K* = 0.89).

The dental caries assessment was done using the DMFT index (12): D is for teeth with dental decay; M is for missing teeth resulting from dental caries; F is for filled teeth. The PUFA index was recorded as follows: P for the involvement of pulp when the pulp chamber orifice was noticeable due to dental caries; U for ulcer following trauma from the sharp parts of a displaced tooth; F for fistula registered in the presence of a sinus tract pus release linked to a pulpally involved tooth; and A for abscess when swelling was present with pus linked to a tooth with pulpal involvement (14). The DAI (15) was used to score malocclusion: (i) normal or minor malocclusion (minimal or no orthodontic treatment required, ≤ 25; (ii) Definite malocclusion (optional orthodontic treatment, 26–30); (iii) Severe malocclusion (orthodontic correction highly desirable, 31–35); (iv) Very severe malocclusion (orthodontic correction is mandatory, >36). TDI was recorded using epidemiological classifications, including the codes of the WHO International Classification of Dental and Stomatology Diseases (15). The scores and codes are as follows: Code 0—no injury (The tooth is sound); Code 1—Treated dental injury (presence of composite restoration, prosthesis replacing missing teeth as a result of trauma); Code 2—enamel fracture only (Trauma confined to enamel); Code 3—enamel dentin fracture (Trauma affecting enamel and dentin); Code 4—pulp injury (Trauma affecting enamel, dentin, and pulp); Code 5—teeth missing due to trauma (tooth avulsed due to trauma only, and not due to dental caries or periodontal disease); Code 9—excluded tooth (Tooth extracted due to dental caries without any signs of dental trauma).

### Statistical Analysis

A one-way ANOVA test was used when three levels or more were categorical, and the response was numerical. A multiple comparison test followed it if the ANOVA test showed a significant difference. The Chi-squared test was applied to assess the correlation between any two categorical variables. The Pearson correlation test was applied to compare the impact of oral health on the OHRQoL among all three groups. The data were analyzed using SPSS version 25.0 statistical software (IBM Inc., Chicago USA), and *p* < 0.05 was significant.

## Results

This study comprised a total of 99 children aged 11–14 years. There were 33 in each group, and the mean ages were foundling, 12.2; delinquent, 13.5; and mainstream, 12.8 years. In each group, the male-female percentage was 57.6 and 42.4 for foundling, 87.9 and 12.1 for delinquent, and 75.8 and 24.2 for the mainstream, respectively (Table 1). Overall scores of DMFT, PUFA, Number of teeth involved in trauma, and OHRQoL scores were summarized in Table 2. The mean total OHRQoL score was lowest in the mainstream and the highest in the delinquent group. Variation in the mean total OHRQoL score was statistically significant between the mainstream and delinquent groups, but no significant difference was observed between the mainstream and foundling or between the foundling and delinquent groups (Table 3). The mean DMFT score was found to be lowest in the mainstream and highest in the delinquent group. The results showed a significant difference in the mean DMFT score among all three. Similarly, the mean PUFA score was lowest in the mainstream and highest in the delinquent group. A statistically significant difference was observed in the mean PUFA score between foundling and delinquent groups and between mainstream and delinquent groups, while this difference was not significant between the foundling and mainstream groups (Table 4). In addition, a statistically significant difference was found in the occlusion status of children in the foundling and delinquent groups, whereas the mainstream group did not show any significant differences. No significant difference was observed in the occlusion status of children among all three groups (Table 5). The presence of TDI was significantly higher in the delinquent in comparison to the other two groups (Table 6). The Pearson correlation test showed that the DMFT, PUFA, and DAI (malocclusion) scores and the TDIs significantly impacted the total OHRQoL score in the foundling and delinquent groups. The DMFT, PUFA, and DAI (malocclusion) score significantly impacted the total OHRQoL score in the mainstream group, but there was no significant impact from TDIs on the total OHRQoL score (Table 7).

**Table 1:** Overall scores of study population.

	**General Information**	**Level**	**Number**	**Percentage**
**General Health**	How do you_evaluate your general health	Bad	9	9.1
		Fair	30	30.3
		Good	60	60.6
	Is there a general practitioner for you	Yes	48	48.5
		No	51	51.5
	Do you usually visit a doctor?	For a periodic medical examination	2	2.0
		To conduct the checks from time to time	1	1.0
		Only when I have a problem	96	97.0
	How difficult is it to find a doctor?	Difficult	5	5.1
		Average	22	22.2
		Easy	72	72.7
	Have you ever been diagnosed by a doctor with a medical condition?	Yes	11	11.1
		No	88	88.9
	Are you using any medicine?	Yes	7	7.1
		No	92	92.9
**Oral Health**	Is there a dentist for you?	Yes	53	53.5
		No	56	46.2
	Do you visit the dental clinic?	For a periodic medical examination	4	4.0
		To conduct the checks from time to time	6	6.1
		Only when I have a problem	89	89.9
	How difficult is it to find a dentist?	Difficult	5	5.1
		Average	19	19.2
		Easy	75	75.8
	Do you brush your teeth?	Daily regularly	35	35.4
		Irregularly	56	56.6
		I don't brush my teeth	8	8.1
**Subjective oral Health**	How to evaluate the health of your mouth?	Excellent	22	22.2
		Very good	39	39.4
		Good	23	23.2
		Are acceptable	7	7.1
		Bad	8	8.1
	How to assess the impact of your oral health on your general health?	Does not affect launch	67	67.7
		Little effect	13	13.1
		Affect sometimes	14	14.1
		Affect greatly	3	3.0
		Always affect	2	2.0
**Occlusion**	Occlusion status	Normal or minor malocclusion	39	39.4
		Definitive malocclusion	36	36.4
		Severe malocclusion	9	9.1
		Very severe malocclusion	15	15.2
**Trauma**	Presence of a dental trauma	No	85	85.9
		Yes	14	14.1

**Table 2:** Overall DMFT, PUFA, and OHRQoL scores, and trauma.

**Details**	**Mean**	**Std. deviation**	**Median**	**IQR**
Age	12.87	1.140	13.00	2.00
DMFT_Decayed	2.12	2.196	2.00	3.00
DMFT_Missing	0.05	0.220	0.00	0.00
DMFT_Filled	0.20	0.534	0.00	0.00
Total DMFT Score	2.37	2.341	2.00	4.00
PUFA_Pulpal_involvement	0.24	0.573	0.00	0.00
PUFA_Ulceration	0.00	0.000	0.00	0.00
PUFA_Fistula	0.06	0.279	0.00	0.00
PUFA_Abscess	0.00	0.000	0.00	0.00
Total PUFA Score	0.30	0.614	0.00	0.00
Total OHRQoL score (Oral symptoms)	5.11	3.886	5.00	6.00
Total OHRQoL score (Functional limitation)	3.98	5.579	2.00	6.00
Total OHRQoL score (Emotional well being)	4.53	6.356	1.00	9.00
Total OHRQoL score (Social well being)	6.15	7.359	3.00	10.00
Total_OHRQoL score	19.76	19.767	13.00	21.00
Number of teeth affected by trauma	0.25	0.644	0.00	0.00

**Table 3:** Comparison of total OHRQoL score in foundling, delinquent and mainstream children groups.

**Group**	***N***	**Mean**	**Std. deviation**	**95% Confidence interval for mean**		***P*-value**
				**Lower bound**	**Upper bound**	
Foundling	33	19.48	24.339	10.85	28.12	0 <0.05
Delinquent	33	30.61	17.704	24.33	36.88	
Mainstream	33	9.18	7.346	6.58	11.79	

^*^Significant.

**Table 4:** The mean prevalence of DMFT and PUFA score in foundling, delinquent and mainstream children groups.

**Descriptives**	**Groups**	**Subjects**	**Mean**	**Std. deviation**	**95% Confidence interval for mean**		***p*-value**
					**Lower bound**	**Upper bound**	
Total DMFT	Foundling	33	2.33	2.056	1.6	0 <0.05	0 <0.05
	Delinquent	33	4.06	2.397	3.21	4.91	
	Mainstream	33	0.73	1.039	0.36	1.1	
Total PUFA	Foundling	33	0.21	0.545	0.02	0 <0.05	0 <0.05
	Delinquent	33	0.64	0.783	0.36	0.91	
	Mainstream	33	0.06	0.242	−0.03	0.15	

^*^Significant; NS = Non-significant.

**Table 5:** The occlusion status in foundling, delinquent and mainstream children groups according to the Dental Aesthetic Index.

**Details**		**Occlusion Status**				***P*-value**
		**Normal or minor malocclusion**	**Definitive malocclusion**	**Severe malocclusion**	**Very severe malocclusion**	
**Foundling**	Count	16	14	1	2	0.00*
	within the group (%)	48.5%	42.4%	3.0%	6.1%	
	Occlusion status (%)	41.0%	38.9%	11.1%	13.3%	
**Delinquent**	Count	15	10	4	4	0.016*
	within the group (%)	45.5%	30.3%	12.1%	12.1%	
	Occlusion status (%)	38.5%	27.8%	44.4%	26.7%	
**Mainstream**	Count	8	12	4	9	0.265^NS^
	within the group (%)	24.2%	36.4%	12.1%	27.3%	
	Occlusion status (%)	20.5%	33.3%	44.4%	60.0%	

^*^Significant; NS = Non-significant.

**Table 6:** The prevalence of dental trauma in foundling, delinquent and mainstream children groups.

**Details**		**Having a dental trauma**		***P*-value**
		**No**	**Yes**	
**Foundling**	Count	30	3	0.00
	within the group (%)	90.9%	9.1%	
**Delinquent**	Count	24	9	0.009
	within the group (%)	72.7%	27.3%	
**Mainstream**	Count	31	2	0.00
	within the group (%)	93.9%	6.1%	

**Table 7:** Correlation between oral findings (DMFT score, PUFA score, occlusion status, dental trauma) and total OHRQoL scores of foundling and delinquent children groups with mainstream children.

**Parameter**	**Univariate linear regression**		**Multiple linear regression**	
	**Coefficient (95% CI)**	***P-*value**	**Coefficient (95% CI)**	***P-*value**
**Total DMFT**	2.34 (1.81, 3.03)	<0.0001*	1.19 (1.11,1.29)	<0.0001*
**Total PUFA**	1.75 (0.92, 3.32)	0.083^NS^		
**Trauma (Number of teeth)**	1.10 (0.31, 3.86)	0.874^NS^		
**Occlusion**	1.91 (1.29, 2.83)	0.001^NS^	1.44 (1.25,1.66)	<0.0001*
**Foundling**	1.01 (0.64, 1.58)	0.977^NS^	1.96 (1.35, 2.86)	0.001*
**Delinquent**	2.83 (1.92, 4.18)	<0.0001*	2.61 (1.70, 4.01)	<0.0001*

*significant; NS, non significant.

## Discussion

There are specific scales specifically designed for the quantitative evaluation of OHRQoL in children and young adults (4, 7) of which CPQ11-14 has been established as a reliable predictor of QoL among children and adolescents (23). Hence, in this study, CPQ11-14 was used to evaluate OHRQoL in children in all groups. Various authors studied the prevalence of dental caries, and a systematic review published in 2017 suggested that in the primary dentition of children in Gulf Cooperation Council Countries, it was 80% (24).

The mean DMFT score for Saudi citizens was found to be 5.38 in primary and 3.34 in permanent dentition in a meta-analysis (25). A systematic review reported a mean DMFT of 5.0 in primary and 3.5 in permanent dentition was observed in Saudi children (8). This study found mean DMFT scores of 2.33, 4.06, and 0.73 for the foundling, delinquent and mainstream groups, respectively, while the mean PUFA scores were 0.21, 0.64, and 0.06, respectively. The mean DMFT and PUFA scores were significantly higher in the delinquent group compared to the mainstream group, suggesting a higher prevalence of dental caries in delinquent compared to mainstream children (26). Pakkhesal et al. (27) concluded that the higher the DMFT score in preschool children, the more negatively impacted OHRQoL. Similar results were reported by Bukhari in working adults (28) and results were not comparable. The results of this study are in accordance with these studies. However, no other studies have mentioned the details of PUFA score assessment and its impact on OHRQoL.

Alongside dental caries and periodontal diseases, malocclusion has also been considered a dental problem (9). Majid (29) suggested that since malocclusion is often accompanied by more significant levels of discontent with appearance, it possibly has a higher negative impact on the OHRQoL. Similar observations were made by Bhatia et al. (30) who found that malocclusion affects boys' emotional well-being, whereas, in girls, it affects the both emotional and social well-being. A similar study on Malay adolescents suggested that the severity of malocclusion is directly proportional to the negative impact on OHRQoL (31). They observed that females manifested a higher negative impact and that the psychological component was predominantly affected. Similar observations were made in this study for all three groups. There was also significant variation in the prevalence of occlusion status of children within the foundling and delinquent groups but no significant difference within the mainstream group.

The PUFA score per person is calculated in the same cumulative way as for the DMFT and represents the number of teeth that meet the PUFA diagnostic criteria. There is no evident association between PUFA and OHRQoL scores found in the present study. These findings are not in agreement with an Indian study where the authors found a positive association between PUFA and OHRQoL (32). It has been well-documented in the literature about the detrimental impact of the presence of dentures, tooth mobility, and edentulism on the oral health-related quality of life (32). Considering the fact that dental caries is a major public health problem among a vast majority of the population, surprisingly there are conflicting results on the impact of the carious lesions on the quality of life of the subjects. Anyhow, these results must be carefully weighed as the study population belonged to different subsets of populations (33–35). This stimulated the investigators of the present study to look into the objective assessment of the complications of untreated carious lesions (PUFA index) on the oral health-related quality of life, especially among a general non-patientt adult rural population in our country. Due to its high psychosocial impact, TDI holds a special position among the causes of a negative OHRQoL (34, 35). Bagchi et al. confirmed that TDI affects a child's school performance and personal relations (36). In this study, TDI was significantly higher in the delinquent group than in the other two but significantly impacted the OHRQoL in all three. Similar observations were made in a 2019 study that found a negative impact of complicated TDI on the OHRQoL on children and their families (37). The impact of DMFT and PUFA on OHRQoL was established among school children with a positive association (38, 39). A Pakistani study conducted among 753 orphan school children reported that 50% of the study population had pulpal involved untreated teeth and also suggested regular visits and initiation of preventive services. Furthermore, recent cross-sectional surveys (40–43) from Saudi Arabia found that parental oral health literacy is critical to maintaining proper oral hygiene and preventive practices (44). The caries burden will be minimized among the children. Based on the present study findings and previous studies' recommendations from Saudi Arabia, the authors opined that it is mandatory to provide regular visits to homes where foundling and delinquent children reside. This will help in minimizing DMFT and eventually the OHRQoL could be improved.

### Strengths and Limitations

This is one of the first studies that explored DMFT, PUFA, TDI, occlusion, and OHRQoL among foundling and delinquent children who resided in Riyadh city, Saudi Arabia to the best of the authors' knowledge. Moreover, these findings could use as a reference for further studies. The small sample size was a limitation of the study, so we recommend a larger sample for future studies. The key variables such as socioeconomic status, social capital, and other structural factors of neighborhoods, and parental education of all groups were not included in the study. These children do not have any of these details, hence were not used in the study. This is also a potential limitation of the study. However, these are the only sample available the Riyadh city, Saudi Arabia. The results of the study may not be generalized due to its limitations.

## Conclusion

With in the study limitations a significant impact on oral health status (DMFT and PUFA indices, malocclusion, and TDI) on the OHRQoL for both foundling and delinquent children compared to mainstream children. However, in the mainstream children, the DMFT and PUFA indices and malocclusion significantly impacted OHRQoL, whereas no significant impact forms TDI was observed. Further studies are waarrent to establish OHRQoL among foundling and delinquent children in Saudi Arabia.

## Data Availability Statement

The raw data supporting the conclusions of this article will be made available by the authors, without undue reservation.

## Ethics Statement

The studies involving human participants were reviewed and approved by the College of Dentistry, King Saud University. Written informed consent to participate in this study was provided by the participants' legal guardian/next of kin.

## Author Contributions

AAA, TA, and AHA involved in the study design, performed the study, written and proof reading of the manuscript, and approved for publication.

## Conflict of Interest

The authors declare that the research was conducted in the absence of any commercial or financial relationships that could be construed as a potential conflict of interest.

## Publisher's Note

All claims expressed in this article are solely those of the authors and do not necessarily represent those of their affiliated organizations, or those of the publisher, the editors and the reviewers. Any product that may be evaluated in this article, or claim that may be made by its manufacturer, is not guaranteed or endorsed by the publisher.
